# Characterization of interfragmentary motion associated with common osteosynthesis devices for rat fracture healing studies

**DOI:** 10.1371/journal.pone.0176735

**Published:** 2017-04-28

**Authors:** Nicholaus Meyers, Matthias Sukopp, Rudolf Jäger, Malte Steiner, Romano Matthys, Bernd Lapatki, Anita Ignatius, Lutz Claes

**Affiliations:** 1 Institute of Orthopedic Research and Biomechanics, Center of Musculoskeletal Research Ulm, University Hospital Ulm, Ulm, Baden-Württemberg, Germany; 2 Department of Orthodontics, University Hospital Ulm, Ulm, Baden-Württemberg, Germany; 3 RISystem AG, Davos, Switzerland; University of Notre Dame, UNITED STATES

## Abstract

Rat models are widely used in preclinical studies investigating fracture healing. The interfragmentary movement at a fracture site is critical to the course of healing and therefore demands definition in order to aptly interpret the experimental results. Estimation of this movement requires knowledge of the fixation stiffness and loading. The characteristic loading for the rat femur has been estimated, but the stiffness of fixation used in rat studies has yet to be fully described. This study aimed to determine the 6 degree of freedom stiffness of four commonly used implants, two external fixators (RatExFix and UlmExFix), a locking plate, and a locking intramedullary nail, in all degrees of freedom and estimate the interfragmentary movement under specific physiological loads. The external fixator systems allow the greatest movement. Mounted 45° anterolateral on the femur, the RatExFix allows an average of 0.88 mm of motion in each anatomic direction while the stiffer UlmExFix allows about 0.6 mm of motion. The nail is far stiffer than the other implants investigated while the plate allows movement of an intermediate magnitude. Both the nail and plate demonstrate higher axial than shear stiffness. The relatively large standard deviations in external fixator shear motion imply strong dependence on bone axis alignment across the gap and the precise orientation of the specimen relative to the loading. The smaller standard deviation associated with the nail and plate results from improved alignment and minimization of the influence of rotational positioning of the specimen due to the reduced implant eccentricity relative to the specimen axis. These results show that the interfragmentary movement is complex and varies significantly between fixation devices but establishes a baseline for the evaluation of the results of different studies.

## Introduction

Murine models are widely used in the investigation of fracture healing, accounting for almost half of all *in vivo* fracture studies [1]. These small animal models afford distinct advantages over larger models. Rodents can be more easily and cheaply housed in large numbers, their short breeding cycles make it possible to utilize large numbers of animals and histologically analyze more time points, and surgical procedures and husbandry can be performed by a single investigator [1, 2]. A disadvantage of these models, however, is that the mechanical properties of the devices used for fracture fixation are not well characterized. This poses a problem since the mechanical environment of the fracture healing zone directly affects the fracture healing process [3–7].

Although the fixation stiffness is frequently explicitly considered and tested in large animal models, to date, little data is published for devices used in small animal models and most studies do not characterize the devices at all [8–14]. This eliminates the possibility of comparing results with experiments using different fixation techniques and limits the scientific value to the internal comparisons made within the study.

The few studies which address the mechanical environment using rat models place emphasis on the axial compression within the fracture gap and disregard the potential shearing motion. For this reason, it is common for researchers to characterize the axial stiffness of their chosen implant system through some sort of uniaxial compression test [15–17]. Some studies have also presented the fixation torsional stiffness [18–20]; however, the confined torsion tests used for the characterization do not provide enough information to predict the complex, 3-dimensional *in vivo* IFM. Although such simplified characterization may hint at the mechanical environment, it also ignores the complicated and potentially highly influential effects of coupled response between the primary loading mode and motion in all other secondary degrees of freedom. For example, the relationship between a force within the anteroposterior plane and the shearing motion response which develops in the medial lateral plane may be significant but would be unquantified and unconsidered without rigorously specifying the 3-dimensional stiffness.

The IFM is a function of the load applied through weight bearing and muscle tension as well as the stiffness of the fracture fixation [3, 21, 22]. The mechanical properties of the fixation devices would be best described by a six-degree of freedom (6DOF) stiffness matrix. Only knowledge of the 6DOF stiffness of the fixation device and the physiological loading of the fractured bone would allow the complete description of the 3-dimensional IFM in the fracture gap, the factor which biomechanically guides the bone healing process.

The aims of this study were therefore to develop, for the first time, the 6DOF stiffness matrices for the most important fixation devices used in rat fracture healing studies as well as present a method by which other researchers may characterize their chosen fracture fixation systems. Since Wehner *et al*. have estimated the physiological loading occurring in the hind limb of the rat throughout gait [22] we have chosen to use their results to estimate the maximum interfragmentary movement (IFM) under four commonly used fixation methods for the basis of comparing the fixation stiffness and mechanical behavior under loading. It is necessary to apply these calculations with respect to the anatomical directions since the physiological loads are expressed with respect to the anatomy. Furthermore, the deformation relative to the anatomical axes is necessary to make the results corporeal for other researchers. Understanding how the choice of fixation affects the complex IFM associated with rat models is a critical step that will allow researchers to more comprehensively analyze their results as well as correlate the results of their studies with those of other researchers.

## Materials and methods

### Fixators

**Fig 1:**
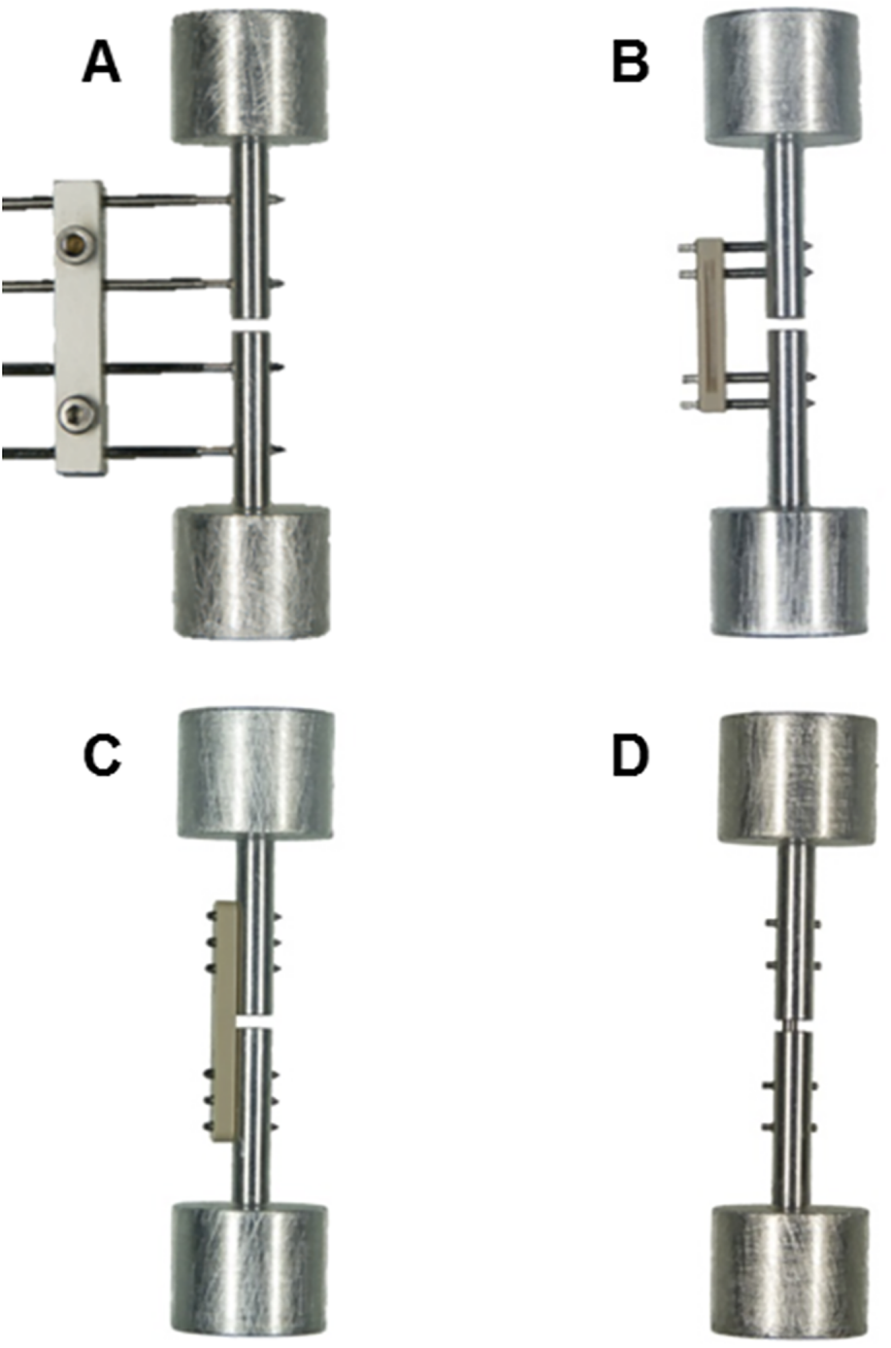
Fixator designs. A) UlmExFix B) RatExFix C) RatFix D) RatNail. The distance between the fixator body and simulated bone surface was set to 12 mm for the adjustable UlmExFix. The remaining fixator designs are standardized and commercially available (RISystem, Davos, Switzerland).

Four different fixator designs commonly used in fracture healing studies employing the rat femur [8–12, 15, 17–19, 23–27] were mechanically characterized in all 6DOF. The first fixation device tested is an adjustable two-component steel and aluminum external fixator that has been used in fracture healing studies at our institute in Ulm [18–20], herein referred to as UlmExFix (Fig 1A). The fixator measures 28.0 mm in length (parallel to bone axis), 5.0 mm in width, and 4.5 mm in depth (direction of mounting screws). The distance between the fixator frame and bone surface is continuously adjustable along the length of the Ø1.2 mm threaded 316L (ISO 5832–1) Kirschner wires (INTERCUS GmbH, Rudolfstadt, Germany) to which it is clamped using two standard M2 screws. The mounting distance, which has a significant influence on the stiffness of the fixation as described by Willie [16], was set to 12 mm in the present study.

The remaining fixator designs are standardized and commercially available research implants (RISystem, Davos, Switzerland). These implants include an external fixator (RatExFix, Fig 1B), an internal locking plate (RatFix, Fig 1C), and a locking intramedullary nail (RatNail, Fig 1D). The bodies of the RatExFix and the RatFix implants are made of PEEK and secured by titanium alloy (TAN; ISO 5832–11) screws. The RatExFix measures 16.5 mm in length, 5.0 mm in width, and 2.5 mm in depth and is mounted approximately 4.5 mm from the surface of the bone. The four mounting screws measure Ø1.0 mm. The RatFix has a length of 23.0 mm, a width of 2.5 mm, a depth of 2.0 mm, and is fixed with six Ø0.7 mm screws. The RatNail is made from implant grade 316L stainless steel and was secured with four locking pins. It measures Ø1.6 mm and has a total length of 39 mm. In the present study, the drilling tip which normally anchors into the proximal femur is not functional, reducing the effective length to 31 mm. The locking pins measure Ø0.8 mm and lock in the near cortex relative to the direction of insertion with a fine thread.

### Specimen preparation

Aluminum tubes with an outer diameter of 3.8 mm, an inner diameter of 2.4 mm, and length of 35 mm with a 1 mm “fracture” gap served as standardized “bones”. Each fixator design was mounted at these tubes across the “fracture“. The aluminum tubes vary only in the exact size and location of the pre-drilled pin/screw holes unique to the individual fixator designs. In order to ensure proper fixation stability, the aluminum tube pin/screw holes were undersized to allow the pins/screws to cut their own threads, thus ensuring maximum purchase. The diameter of the pre-drilled holes was 0.85 mm for the RatExFix, 0.95 mm for the UlmExFix, 0.65 mm for the RatFix plate, and 0.70 mm for the RatNail locking screws. Six specimens were tested for each fixator design and no specimen mounts were reused.

### Test set-up and data collection

**Fig 2:**
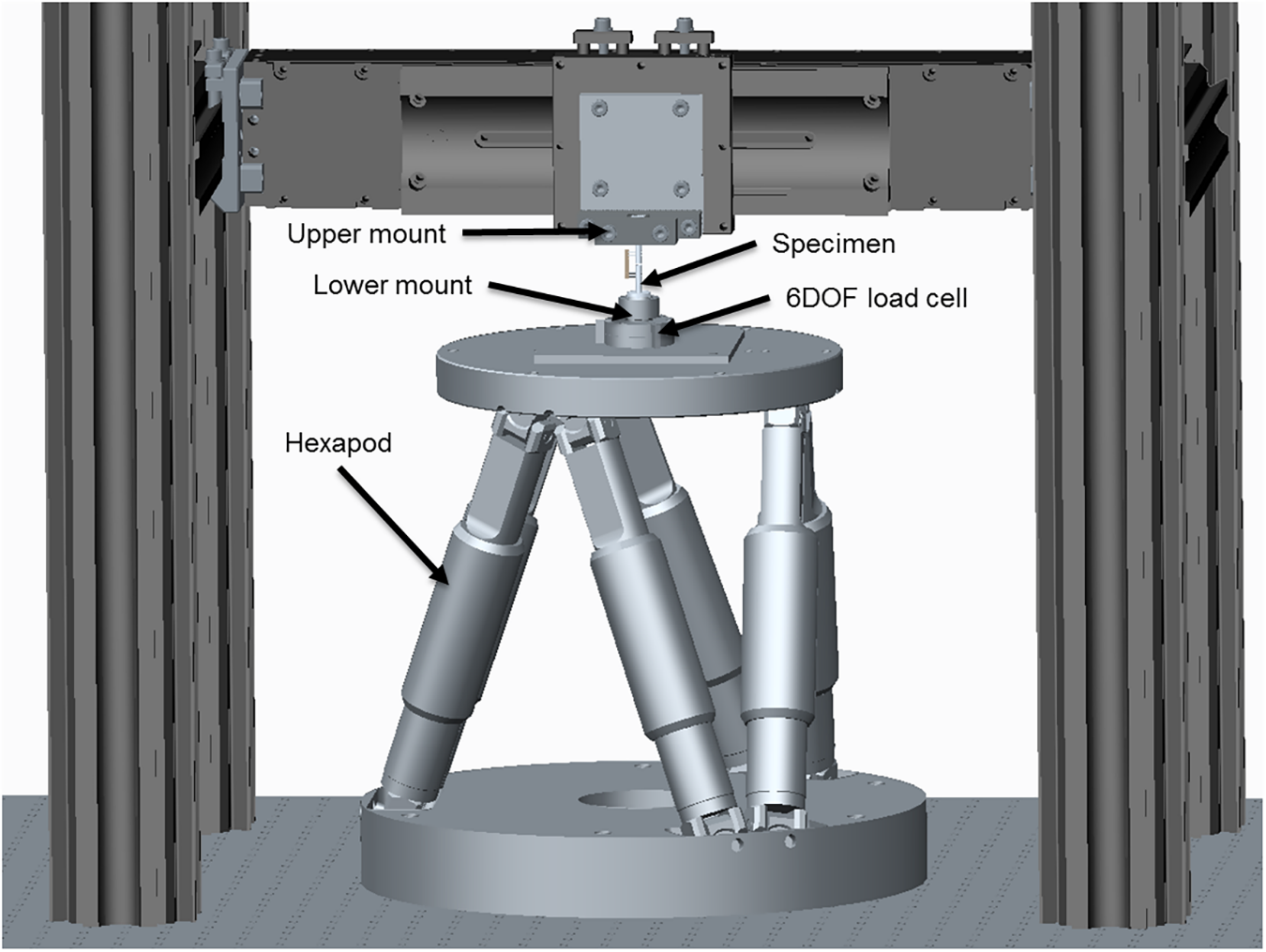
Hexapod with mounted specimen.

**Fig 3:**
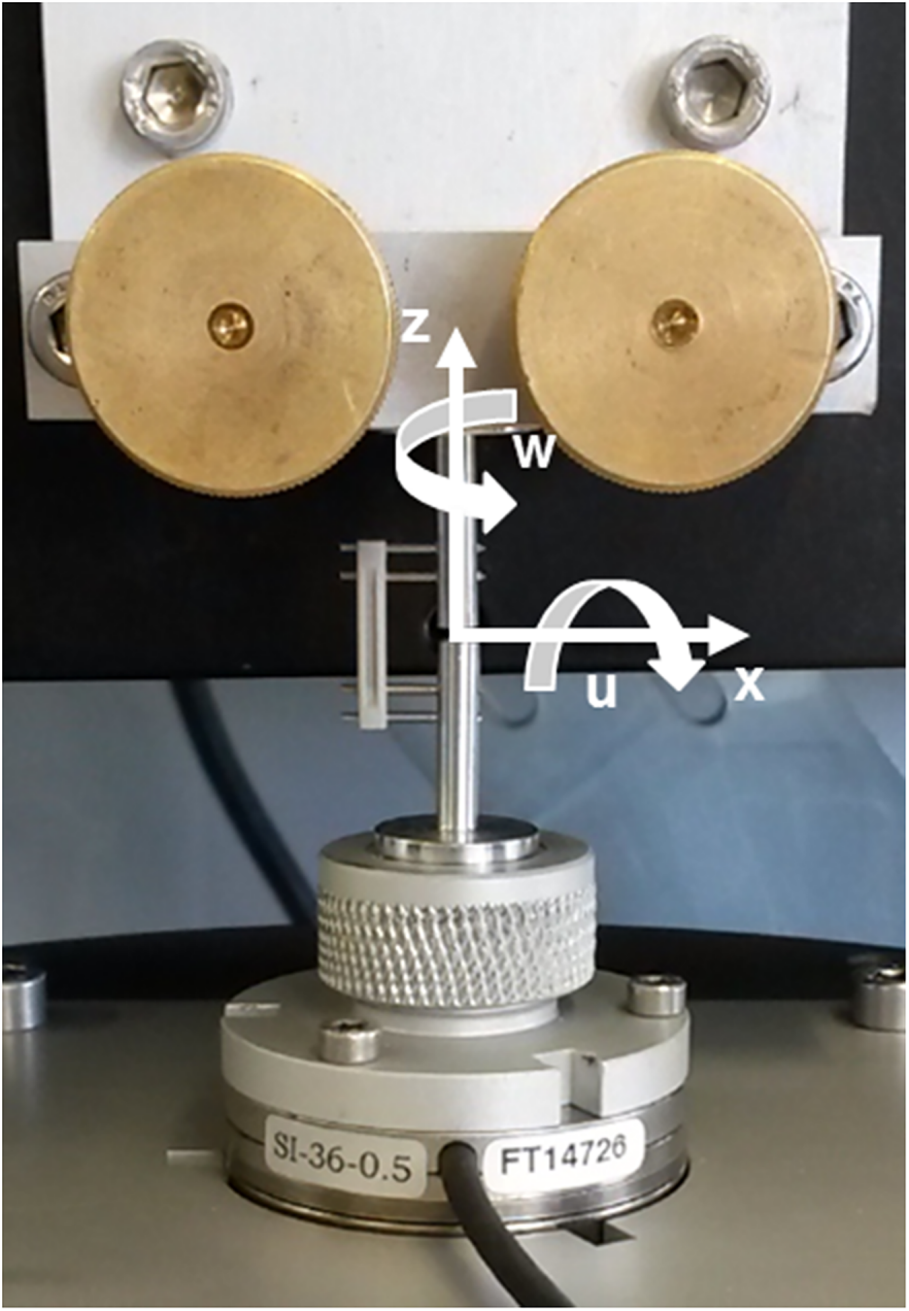
Coordinate system used for independent load cases. The y-axis is mutually perpendicular to the x and z axes shown and points into the page away from the reader. The rotations u and w are show as rotations about the x and z axes respectively. Rotation about the y-axis follows the right hand rule and is signified by v.

**Fig 4:**
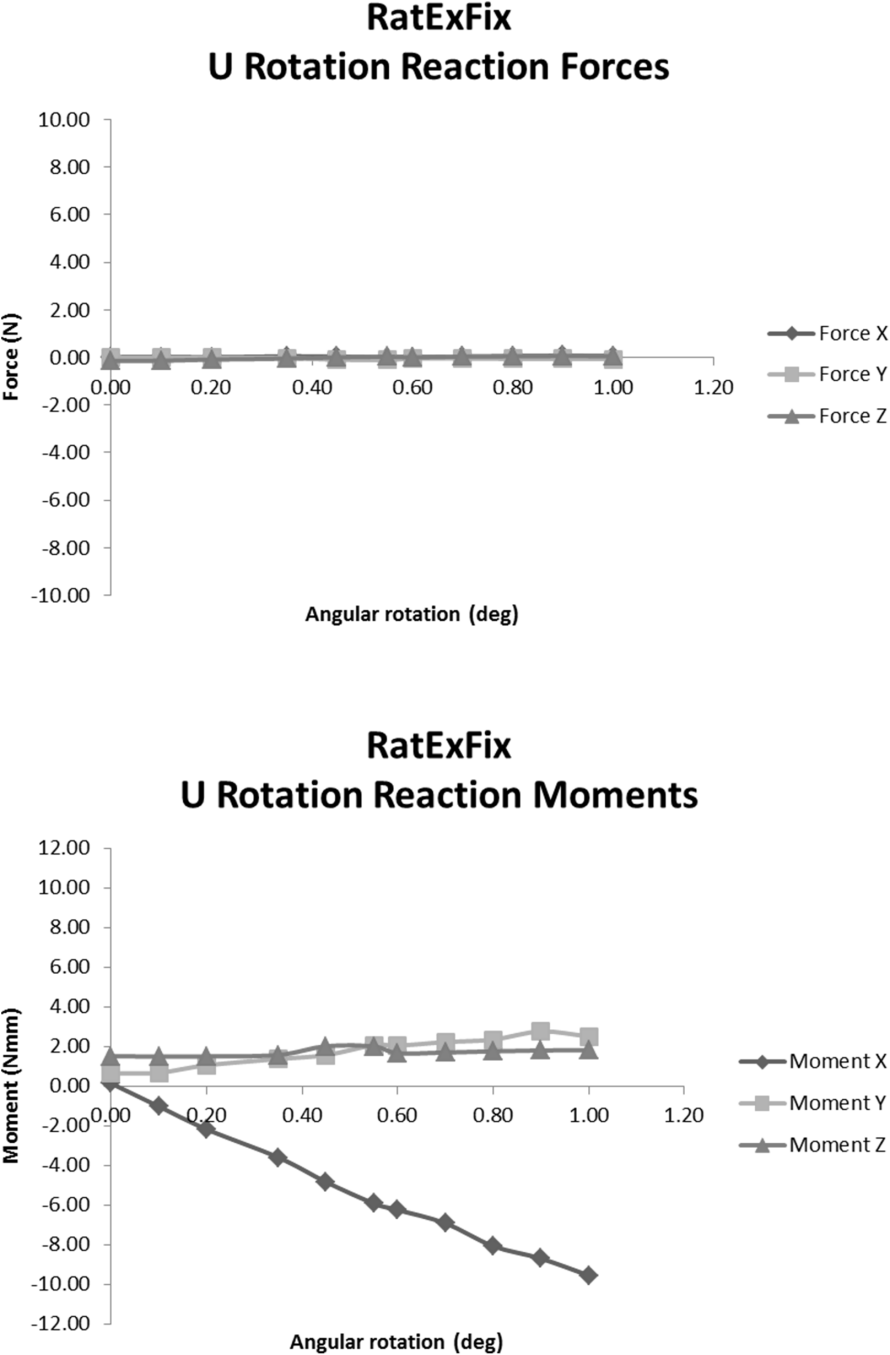
Example loading response curves. Example force (top) and moment (bottom) response curves to applied rotation, u, about the x axis for the RatExFix implant.

The specimens were rigidly fixed into an H-850 6-Axis Hexapod Microrobot (Physik Instrumente (PI) GmbH & Co. KG, Karlsruhe, Germany) using custom made mounts. The upper aluminum fixator mount was clamped into the immobile frame surrounding the Hexapod. The lower aluminum fixator mount is radially clamped within a fixture directly attached to a 6 DOF FT-Nano 43 load cell (ATI Industrial Automation, Inc., Apex, North Carolina, United States) (Fig 2). For each specimen, the hexapod was electronically positioned to eliminate clamping preload thereby establishing the home position for the specimen. The specimen was subsequently subjected to six separate load cases under precise displacement control using LabVIEW control software developed in-house. Single axis translations along each axis of a Cartesian coordinate system constitute three of the six load cases. Non-contemporary rotations about each of these axes comprise the remaining three independent load cases (Fig 3). Each load case was applied and returned to the home position over three cycles to minimize contact settling; the data was collected during the final cycle. The reaction forces and moments were recorded in all 6 DOF providing information on the coupling of motion with forces and moments in all directions, critical for a complete understanding of the mechanical behavior under complex loading as would occur during gait. The magnitude of applied translation or rotation was determined by the primary reaction force or moment measured by the 6DOF load cell. Incremental translations and rotations were made until the physiological loading regime established by Wehner et al. had been surpassed. Therefore the magnitude of motion depended on the fixator design and direction of loading. Furthermore, the magnitude of motion was allowed to vary between specimens of the same fixator type as necessary to surpass the physiological loading regime. This was done to rudimentarily approximate the *in vivo* conditions as closely as permissible within the constraints of the *in vitro* testing set-up. Using the hexapod, we were able to apply pure bending around the center point of the “fracture” gap of each specimen whereas similar studies require the application of a bending moment through a cantilever configuration [28, 29] which makes isolation of the effects lateral translation and bending modes difficult. A coordinate transformation was applied to the force and moment reactions measured beneath the specimens through the motion control software to provide the virtual forces and moments at the center of the fracture gap. This allows the establishment of a base of comparison with the mid-diaphysis physiological forces and moments predicted by Wehner *et al*. An example of the reaction forces and moments in response to an applied angular displacement around the x axis for the RatExFix is given in Fig 4.

### Constitutive relationship

The following analysis assumes each fixator behaves in a linear elastic manner within the applied loading regime in accordance with previous studies characterizing the 6DOF stiffness of fixation [28, 29]. The data collected for each fixator design was used to calculate stiffness matrices using the method presented by Gardner and Weemaes [29]. The kinematic response of the implant system under the action of a load or combination of loads can be characterized by the constitutive relationship:

$$K_{ij}U_{j}=R_{i}$$ (1)

in which K_ij_ is a representative element of a stiffness matrix in the i^th^ row and j^th^ column, U_j_ is the j^th^ element of a column displacement vector acting on the system relative to a chosen coordinate system, and R_i_ is the i^th^ element of the column vector of the reaction forces and moments. Note that the Einstein summation convention is applied to dummy indices.

$$\left[\begin{array}{cccccc} K_{xx} & K_{xy} & K_{xz} & K_{xu} & K_{xv} & K_{xw}\\ K_{yx} & K_{yy} & K_{yz} & K_{yu} & K_{yv} & K_{yw}\\ K_{zx} & K_{zy} & K_{zz} & K_{zu} & K_{zv} & K_{zw}\\ K_{ux} & K_{uy} & K_{uz} & K_{uu} & K_{uv} & K_{uw}\\ K_{vx} & K_{vy} & K_{vz} & K_{vu} & K_{vv} & K_{vw}\\ K_{wx} & K_{wy} & K_{wz} & K_{wu} & K_{wu} & K_{ww} \end{array}\right]\left[\begin{array}{c} \delta_{x}\\ \delta_{y}\\ \delta_{z}\\ \gamma_{u}\\ \gamma_{v}\\ \gamma_{w} \end{array}\right]=\left[\begin{array}{c} F_{x}\\ F_{y}\\ F_{z}\\ M_{u}\\ M_{v}\\ M_{w} \end{array}\right]$$ (2)

Eq 2 depicts the full matrix representation in which δ corresponds to a translation in millimeters (mm) and γ corresponds to a rotation in degrees (°) about the axis indicated by its respective subscript. The subscripts x, y, and z indicate the axis along which the translations are made and the reaction forces develop. The subscripts u, v, and w correspond to rotation or reaction moment about the x, y, and z axes, respectively. The reaction forces are indicated by F and measured in Newtons (N) while the reaction moments are indicated by M and measured in Newton-millimeters (Nmm). The elements of the stiffness matrix correspond to the relationship between motion in direction i and the corresponding force or moment reaction j. For example, stiffness element K_xv_ would correlate the force which develops along the x axis due to a rotation applied about the y axis. The units of each element of the stiffness matrix are determined by the constitutive coupling it represents. Let **T** represent any subscript x, y, or z and **R** represent any subscript u, v, or w. Elements of the form K_**TT**_ will have units of N/mm. Elements of the form K_**TR**_ will have units of N/degree. Elements of the form K_**RT**_ will have units of Nmm/mm. Elements of the form K_**RR**_ will have units of Nmm/degree. Taking our previous example, element K_xv_ will have the units of N/degree since it maps a reaction force to a rotational displacement.

A typical load case will be represented by a six element vector of displacement and rotation values, only one of which will be non-zero. A single load case in which all but a single displacement or rotation is non-zero will therefore provide a system of six algebraic equations which can be solved directly to determine six of the unknown stiffness values. In order to solve for all 36 of the stiffness values, we must therefore apply six independent load cases.

### Augmentation

Eq 1 can be augmented to include all six of the independent load cases directly and can be expressed by

$$K_{ik}U_{kj}=R_{ij}$$ (3)

in which each column j of U_kj_ references an independent load case and each column j of R_ij_ refers to the reaction forces and moments which develop in response to load case j of U_kj_. The matrix representation thus appears as

$$\left[\begin{array}{cccccc} K_{xx} & K_{xy} & K_{xz} & K_{xu} & K_{xv} & K_{xw}\\ K_{yx} & K_{yy} & K_{yz} & K_{yu} & K_{yv} & K_{yw}\\ K_{zx} & K_{zy} & K_{zz} & K_{zu} & K_{zv} & K_{zw}\\ K_{ux} & K_{uy} & K_{uz} & K_{uu} & K_{uv} & K_{uw}\\ K_{vx} & K_{vy} & K_{vz} & K_{vu} & K_{vv} & K_{vw}\\ K_{wx} & K_{wy} & K_{wz} & K_{wu} & K_{wu} & K_{ww} \end{array}\right]\left[\begin{array}{cccccc} \delta_{x} & 0 & 0 & 0 & 0 & 0\\ 0 & \delta_{y} & 0 & 0 & 0 & 0\\ 0 & 0 & \delta_{z} & 0 & 0 & 0\\ 0 & 0 & 0 & \gamma_{u} & 0 & 0\\ 0 & 0 & 0 & 0 & \gamma_{v} & 0\\ 0 & 0 & 0 & 0 & 0 & \gamma_{w} \end{array}\right]=\left[\begin{array}{cccccc} F_{x,1} & F_{x,2} & F_{x,3} & F_{x,4} & F_{x,5} & F_{x,6}\\ F_{y,1} & F_{y,2} & F_{y,3} & F_{y,4} & F_{y,5} & F_{y,6}\\ F_{z,1} & F_{z,2} & F_{z,3} & F_{z,4} & F_{z,5} & F_{z,6}\\ M_{u,1} & M_{u,2} & M_{u,3} & M_{u,4} & M_{u,5} & M_{u,6}\\ M_{v,1} & M_{v,2} & M_{v,3} & M_{v,4} & M_{v,5} & M_{v,6}\\ M_{w,1} & M_{w,2} & M_{w,3} & M_{w,4} & M_{w,5} & M_{w,6} \end{array}\right]$$ (4)

in which the numeric subscript of matrix R_ij_ references the specific load case where 1–6 indicate the columns of matrix U_kj_. This system maps each of the 36 unknown stiffness values to a specific force or moment as the result of an applied translational or rotational displacement. This system of equations was solved by post-multiplying R_ij_ with the inverse of U_kj_ using MATLAB software (MathWorks, Inc., Natick, Massachusetts, United States).

### Flexibility matrices

The resulting matrices give the forward relationship mapping an applied IFM to the virtual reaction forces and moments within the fracture gap. In order to make this data useful for the prediction of IFM, the calculated stiffness matrices must be inverted to give flexibility matrices which map a vector of physiological loads to an expected IFM at the fracture gap. This is accomplished by matrix inversion and was calculated using the inversion function of MATLAB.

### Interfragmentary motion in the anatomical directions

**Fig 5:**
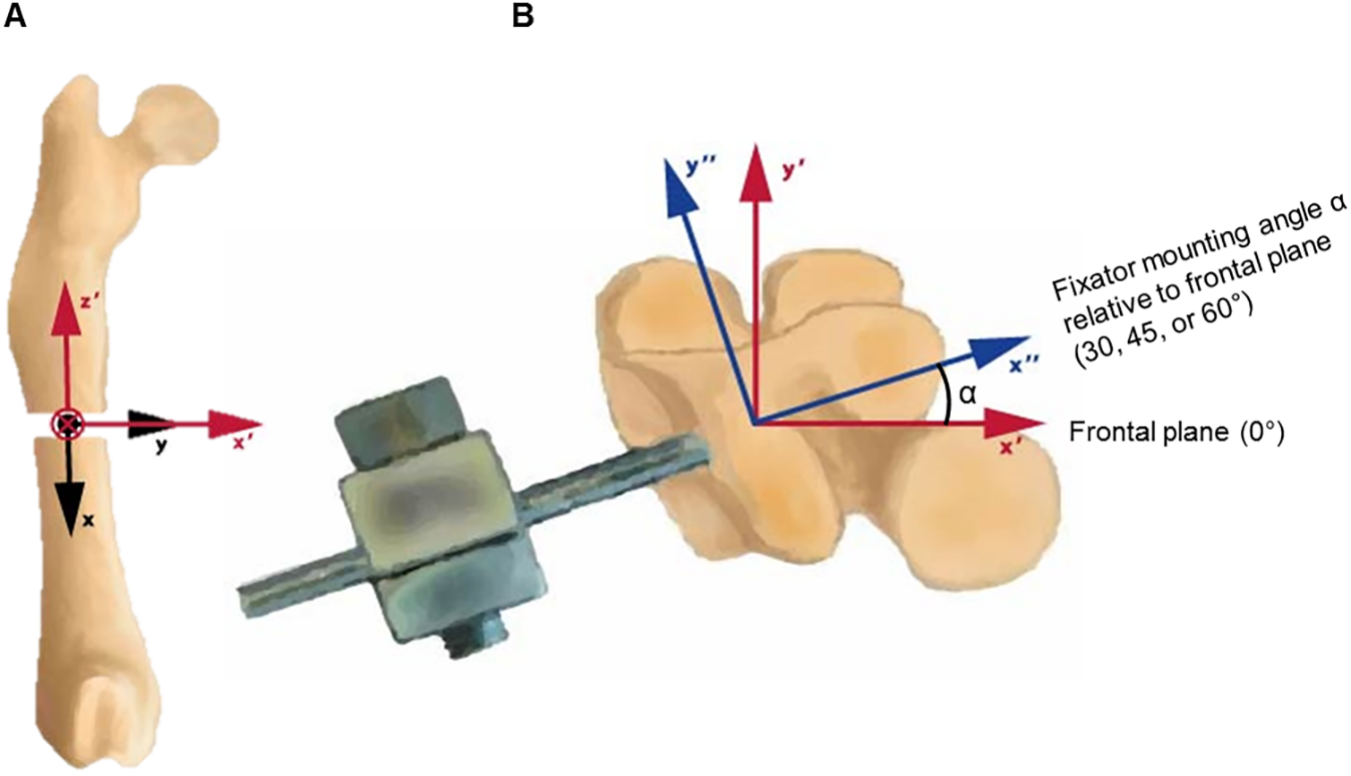
Coordinate system transformations. In order to transfer the data collected in the hexapod experiments to the *in* vivo application of fixation relative to the anatomic directions, it is necessary to perform the following coordinate transformations. A) First coordinate system transformation from Wehner et al. to hexapod coordinate orientation B) Cranial view: second coordinate system transformation accounting for the loading relative to the fixator through an angle α. The physiological loads presented by Wehner et al. in the xyz coordinate system have been transformed to the x”y”z” coordinate system for α equals 30, 45, and 60°.

In order to calculate the IFM at the fracture site along the anatomical directions, it was first necessary to perform coordinate transformations on the physiological loads as defined by Wehner *et al*. The first coordinate transformation simply reoriented the coordinate system of Wehner *et al*. to the coordinate system established for the testing of the implants (Fig 5A). The loads applied for calculation of the IFM were the maximum values calculated by Wehner et al. over the course of the gait cycle at the mid diaphysis of the femur and are given as follows relative to the hexapod coordinate system (Fig 3).

$$\left[\begin{array}{c} F_{x}\\ F_{y}\\ F_{z}\\ M_{x}\\ M_{y}\\ M_{z} \end{array}\right]=\left[\begin{array}{c} -0.9\\ 0.6\\ -6\\ 10.7\\ 6\\ 5 \end{array}\right]*BW\approx \left[\begin{array}{c} -3\;N\\ 2\;N\\ -19\;N\\ 34\;Nm\\ 19\;Nm\\ 16\;Nm \end{array}\right]$$ (5)

Here, BW indicates the body weight of the rat. In the case of the rat analyzed by Wehner et al. the body mass was 325 g, approximately 3.2 N. This vector would apply to a fixator mounted in the mediolateral plane. A second series of standard coordinate transformations equivalent to a rotation about the z axis (corresponding to the long axis of the femur) were applied to this basis physiological load vector for calculation of the IFM with the fixator mounted at each 30, 45, and 60°. The transformed vectors correspond to the loading that would be acting on the fixator relative to the coordinate system used to calculate the stiffness and flexibility matrices (Fig 5B). These angles were chosen to encompass the typical range through which a fixator may be implanted during surgery. From these adjusted physiological load vectors and the flexibility matrices, the IFM could be calculated as motion along or about the axes defined for the specimen testing procedure. In order to report the IFM occurring within the anatomical directions (anteroposterior: A-P, mediolateral: M-L, axial), a final coordinate transformation was applied to the resulting IFM vectors to return the representation to the physiological coordinate system (Fig 5A), essentially reversing the rotation applied to the physiological load.

In calculating the stiffness matrices from the collected data, it was found that certain components of the mathematical sign of certain components of the stiffness matrices varied between specimens of the same fixation. This can be explained by the variability of implantation and the resulting angular deviation and offset displacement between the proximal and distal ends of the specimen. Therefore, the IFM is reported as an average of the absolute value of the motion within a specific anatomic plane for each specimen. This is a valid approach as it is the magnitude and mode (shear, compression, tension, torsion) which dictates the healing response independent of the exact direction of motion (medial vs lateral, anterior vs. posterior).

### Statistical analysis

**Fig 6:**
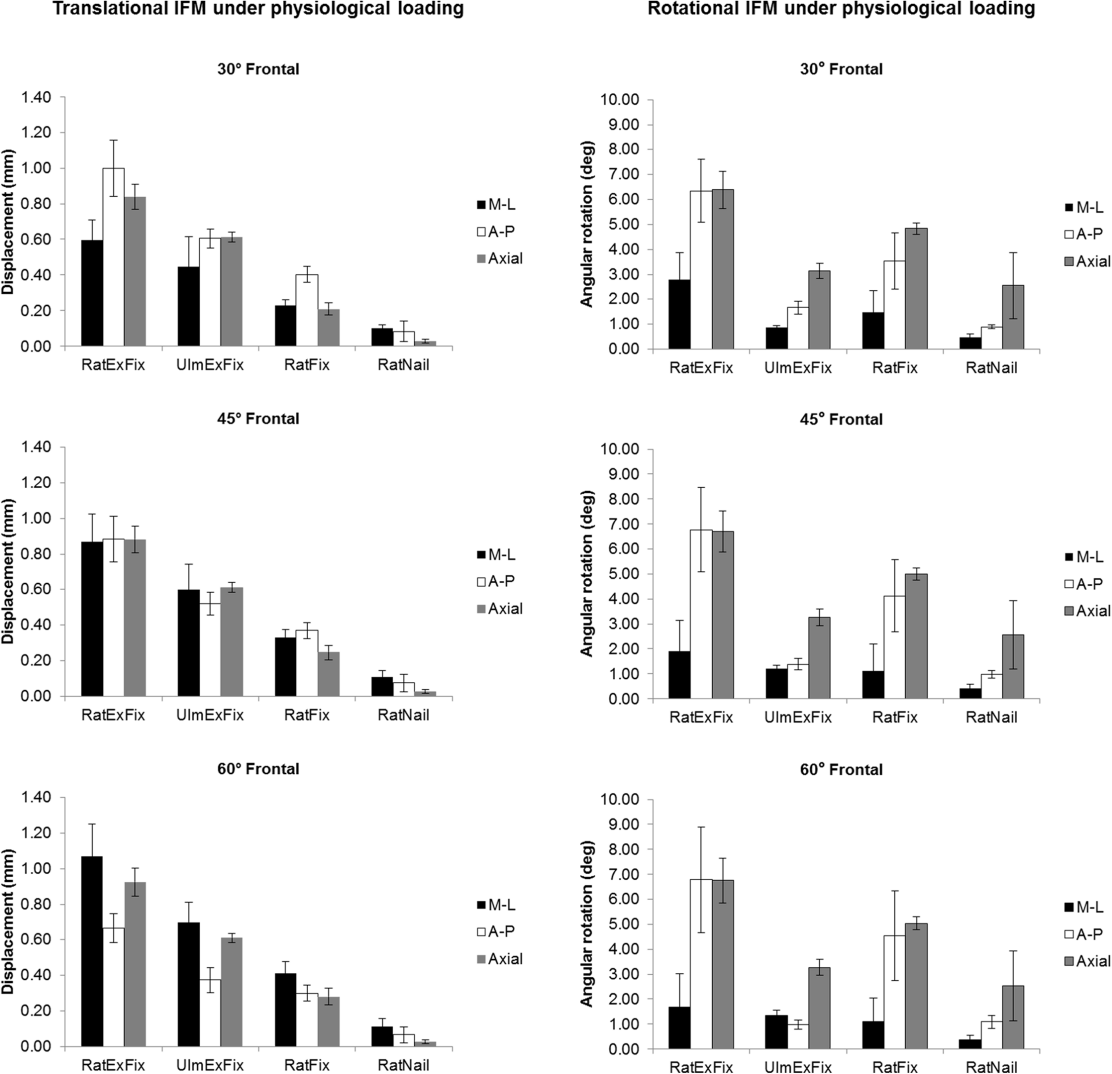
Translational and rotational IFM under physiological loading, organized by fixator. Translational (left) and rotational IFM (right) across 30, 45, and 60° fixator mounting relative to the frontal plane for each fixation design presented as the mean value of IFM ± standard deviation. *p<0.05.

**Fig 7:**
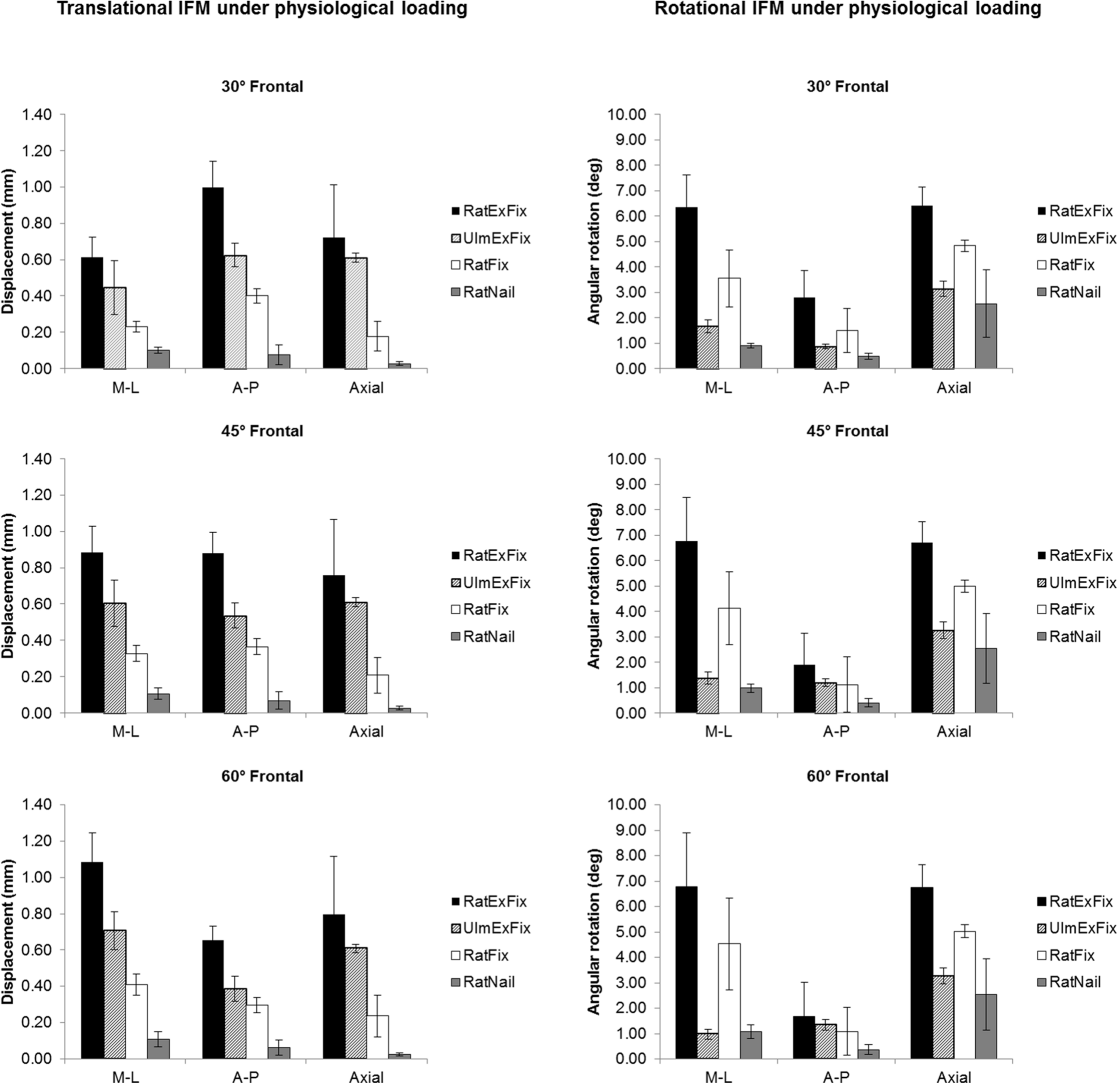
Translational and rotational IFM under physiological loading, organized by anatomic direction. Translational (left) and rotational IFM (right) across 30, 45, and 60° fixator mounting relative to the frontal plane for each anatomical direction presented as the mean value of IFM ± standard deviation. *p<0.05.

The maximum translational and rotational physiological IFM (Figs 6 and 7) were calculated for implants positioned at 30, 45, and 60° anterolaterally relative to the frontal plane (Fig 5B). A normal distribution of data could not be assumed and a test for normal distribution would be questionable with the small number of samples used for this study. Therefore, a Kruskal-Wallis test with Bonferroni correction was performed for A-P translation for each fixator design mounted at 45°. The significance level α was set to 5%. The large number of possible tests was restricted to A-P translation in the 45˚ plane of implant application because this is likely the most critical IFM with respect to bone healing in the most commonly implanted position. Since the data is not independent, further statistical tests would increase the p-value often eliminating the significance due to the Bonferroni correction that was performed for multiples testing.

## Results

A comparison of the A-P translation for each fixator indicates that the RatExFix is significantly different from both the RatNail (p < 0.001) and the RatFix (p = 0.020) while the UlmExFix allows significantly greater motion than the RatNail (p = 0.020).

### RatExFix

The RatExFix is the most compliant implant tested allowing an average initial axial motion of 0.84 ± 0.07, 0.88 ± 0.08, and 0.92 ± 0.08 mm of motion for 30, 45, and 60° anterolateral implantation, respectively. When mounted at 45° the magnitude of IFM is nearly equal in all anatomical directions, allowing an average of 0.88 mm of mediolateral (M-L), anteroposterior (A-P), and axial interfragmentary translation. When mounted at 30°, the peak A-P translation exceeds the axial IFM while the M-L translation is reduced. When mounted at 60°, a similar trend is observed with the M-L translation exceeding the axial motion and the A-P translation decreasing. The axial IFM is much more consistent than either the M-L or A-P translation across all mounting planes.

For all mounting positions, the A-P bending (rotation within sagittal plane) and axial rotation are nearly equal with approximately 7° of motion. The M-L bending (rotation within frontal plane) is markedly lower in all cases (Figs 6 and 7).

### UlmExFix

When the fixator frame is mounted 12 mm from the surface of the bone, the UlmExFix is stiffer than the RatExFix but demonstrates similar trends across the mounting angles. The axial IFM is more consistent than for the RatExFix, measuring 0.61 ± 0.03 mm in every mounting angle, and when mounted at 30°, the A-P translation is equal to the axial motion. When mounted at 60°, the M-L translation exceeds the axial IFM. The axial rotational stiffness and the A-P bending stiffness are much higher for the UlmExFix than for the RatExFix at every mounting angle. Notably, the axial rotational motion is nearly twice that of the A-P or M-L bending motion for this fixation system (Figs 6 and 7).

### RatFix

The translational stiffness of the RatFix is greater than either of the external fixator systems, but it is more compliant in axial rotation and A-P bending than the UlmExFix. Of interest, when mounted in the 30° position, the A-P translation is approximately twice the M-L translation and axial IFM. The M-L bending motion tends to be equal to or greater than the M-L bending of the UlmExFix, however, the standard deviation is relatively large in this direction, averaging ±1°. Furthermore, the pattern of rotational motion is more similar to RatExFix than the UlmExFix is to the RatExFix (Figs 6 and 7).

### RatNail

The RatNail is, by far, the stiffest implant system investigated allowing an average of only 0.03 ± 0.01mm of axial IFM. For each calculated mounting angle, the average M-L translation was greatest, followed closely by A-P translation. However, the M-L and A-P translation demonstrated larger standard deviations than the axial IFM. In contrast, the average amount of axial rotation was much greater than either A-P or M-L bending but with a standard deviation larger than ±1.3°, nearly an order of magnitude greater than the deviation in the bending modes (Figs 6 and 7).

## Discussion

This study presents a method for the calculation of a 6DOF stiffness matrix for the most important fracture fixation devices used in rat models. Using the musculoskeletal model of Wehner et al., the IFM in the fracture gap could be estimated for use as a basis of comparison of fixation behavior. The results demonstrate that the IFM is always a combined and complex movement that also depends on the anatomical location of the fixation device.

Typical confined torsion testing of implant constructs yields little useful information because the *in vitro* boundary conditions do not correspond to the *in vivo* boundary conditions. Because the complex relationships between displacements and reaction forces and moments are not defined, one cannot mathematically compensate for the variation in boundary conditions. Although this study applied confined torsion, the reactions are recorded in all 6DOF and the coupling of displacements with the 6 DOF forces and moments in the other five load cases provide a comprehensive model freeing it from the boundary conditions applied during testing. This information is vital; literature indicates that shearing motion may inhibit bone healing [6, 27], so large A-P and M-L motion may not be ideal and may have a significant impact on studies using different fixation techniques. It is noteworthy that none of the implants are stable in the lateral directions relative to the amount of axial motion they permit, respectively. That is to say, the magnitude of lateral motion typically meets or exceeds the axial IFM potentially shifting the dominating stimulus to an unexpected mode.

### RatExFix and UlmExFix

The larger standard deviations associated with A-P and M-L shear in the external fixators and variation in the sign of respective stiffness matrix components of different samples of the same fixator indicate strong dependence on a few variables including alignment of the femoral axis across the fracture gap and the precise angular position of the fixation relative to the directions of applied load during testing. The latter is analogous to varying rotational positioning about the femoral axis and is the likely cause of sign discrepancy. If the direction of loading varies relative to fixation plane, the reaction forces and moments will vary accordingly due to the “anisotropic” structural stiffness. Furthermore, for the RatExFix, measurements of our specimens indicate that the actual mounting distance from the bone varies between 3.8 and 4.5 mm using the recommended mounting technique. These factors also help explain the much smaller standard deviations associated with the RatNail and RatFix systems in which the implant is coincident with and minorly eccentric to the specimen axis, respectively. This improves the reproducibility of alignment across the fracture gap compared to the large eccentric positioning of the external fixators and reduces the variability in structural stiffness resulting from small angular deviations relative to the loading direction.

The two external fixators allow very large translational motion in every plane relative to the typical gap size used in fracture healing experiments employing the rat. A 1 mm osteotomy stabilized with this implant will lead to an average initial axial strain of 84 ± 7, 88 ± 8, and 92 ± 8%. Strains of this magnitude may delay healing relative to optimal fixation stiffness. Furthermore, it may illicit a biochemical response that would not correspond well to studies using other, more stable fixation techniques. Importantly, it is known that certain biological factors have a biphasic dose response [30, 31]. The very high strain may lead to disproportionately high or low concentrations of biological factors [32, 33] leading to false conclusions pertaining to their clinical influence. In this way, two studies differing only in choice of fixation may report conflicting results.

In the present study, the UlmExFix is visibly stiffer than the RatExFix (Figs 6 and 7), however no statistically significant difference was found between A-P translation for these two external fixators when mounted at 45° (p = 0.850). However, unlike the RatExFix, the fixation stiffness of the UlmExFix can be increased continuously by reducing the distance between the fixator body and bone from the flexible configuration used in this study [16] leading to lower IFM and corresponding IFS. Understanding the precise 3-dimensional repercussions of such implantation adjustments would require equivalent testing at other distances but would potentially make the difference in mechanical behavior between the external fixators significant.

### RatFix

The RatFix is intermediate in its stiffness characteristics and has relatively small standard deviations across all translational DOFs. However it is more flexible, in torsion and the bending modes than the UlmExFix and the RatNail, likely due to the PEEK material from which it is made in contrast to the metal construction of the two other implants.

### RatNail

The very stiff RatNail fixation is due to the combination of its unusually large diameter in comparison to traditional Kirschner wire intramedullary fixation as well as its locking feature provided by the four locking screws which pass through the bone and nail. These features are in stark contrast to the typical K-wire internal fixation used in many rat fracture studies [34–37], which are inherently flexible, unstable, and give nearly no resistance in torsion thereby leading to large IFM [2, 38]. The A-P and M-L IFM are higher than the axial IFM and is likely a result of the slight toggling allowed between the screws and the nail. The locking screws prevent contact between nail and the canal of the aluminum testing mounts used in this study, however, such contact within the medullary canal may add stability to the construct *in vivo*. Additionally, the loss of functionality of the proximal anchoring thread would likely contribute to a larger lateral toggle than that present *in vivo*.

### Limitations

The method for measuring implant flexibility and the resulting IFM trends under loading are independent of the exact physiological load. However, the physiological loading vector is based on values determined by inverse dynamic analysis for a single specific rat. *In vivo* conditions will vary greatly between individual specimens based on exact bone morphology, pain tolerance, weight, and implantation variables. For this reason, the resulting IFM magnitudes should not be taken as precise values. Furthermore, the predicted motion does not consider the potential difference between fixation stiffness of screws in aluminum tubes rather than bones and the stabilization resulting from resistance due to surrounding soft tissue and the callus, or the decreased post-surgical weight bearing.

This study assumes that a linear elastic coupling exists between the load in any given degree of freedom and the resulting kinematic response in any other. We have checked the validity of this assumption by plotting the incremental motion steps against each of the six reaction force and moment values corresponding to the respective position steps to assess the linearity of every force and moment response with respect to motion in each degree of freedom. An example of this is given in Fig 4. The results indicate that there is a strong linear correlation for many of the cases, but that the assumption does not generally hold. The presence and degree of nonlinearity varies between fixation type and between individual specimens of the same fixation. The latter case further indicates that implantation technique plays an important role in the mechanical behavior of the fixation under load; angular misalignment and displacement of the proximal bone segment relative to the distal segment may alter the IFM at the fracture site. In particular, the RatNail consistently demonstrated significant nonlinearity. This indicates that the intramedullary fixation may be the most sensitive to implantation technique. Because the linearity assumption does not hold in all cases for all coupled kinematic responses, the analysis performed would not apply to other loading cases. Regardless, we do not feel the cases of nonlinearity reduce the value of the present work. The calculations are meant to demonstrate the general relative behavior of each implant. In the present study, data collection was performed to closely match the reaction force and moment response with the maximum physiological loads calculated by Wehner et al. This ensured that the calculations of IFM used to compare fixation would correlate with the collected data and negate any nonlinearity between the unloaded condition and the testing conditions by reducing interpolation. We reiterate that such an analysis cannot, and is not intended, to be used as a predictive model of precise *in vivo* IFM values due to the system nonlinearities and variations as well as the large discrepancy in physiological loading across individuals resulting from body weight variation, morphological variation, post-surgical pain tolerance, and implantation variations.

## Conclusions

In order to compare and understand the results of different fracture healing studies, the experimental conditions must also be understood. The results of this study show that the IFM associated with physiological loads is complex and varies significantly between commonly used rat fixation devices and their application relative to anatomical planes. Studies which characterized the stiffness of the fracture fixation in only one loading direction missed information regarding the IFM in the other loading planes and may have led to the misinterpretation of results. In addition, this study establishes a baseline for the evaluation of the results of different studies. This study adds to the body of data and understanding of research implant behavior allowing researchers using any of these particular systems to better understand their experimental set-up. Furthermore, it provides a general method and encouragement for researchers to characterize and publish complete implant stiffness data for whichever fixation system and technique they employ.

By including a full mathematical description of the behavior of fixation systems, researchers will be able to mine the work of others more efficiently and potentially draw important conclusion that are currently obscured by undefined mechanics thereby increasing the utility of experimental results. This characterization can be completed at any time, and may potentially help uncover new information useful for the improvement of experimental design for future studies.

## Supporting information

S1 Dataset All data collected for each fixator and specimen is available within the accompanying supporting dataset. (ZIP)
